# Malnutrition management in children with chronic kidney disease

**DOI:** 10.1007/s00467-024-06436-z

**Published:** 2024-07-02

**Authors:** Antonio Corsello, Chiara Maria Trovato, Valeria Dipasquale, Emanuele Proverbio, Gregorio Paolo Milani, Antonella Diamanti, Carlo Agostoni, Claudio Romano

**Affiliations:** 1 Department of Clinical Sciences and Community Health, University of Milan, Milan, Italy; 2 Pediatric Unit, Fondazione IRCCS Ca’ Granda Ospedale Maggiore Policlinico, Milan, Italy; 3 Hepatology Gastroenterology and Nutrition Unit, Bambino Gesù Children Hospital, Rome, Italy; 4 Pediatric Gastroenterology and Cystic Fibrosis Unit, Department of Human Pathology in Adulthood and Childhood “G. Barresi”, University of Messina, Messina, Italy

**Keywords:** Pediatric chronic kidney disease, Nutritional management, Protein energy wasting, Enteral nutrition, CKD, Gastrostomy

## Abstract

Chronic kidney disease (CKD) encompasses diverse conditions such as congenital anomalies, glomerulonephritis, and hereditary nephropathies, necessitating individualized nutritional interventions. Early detection is pivotal due to the heightened risk of adverse outcomes, including compromised growth and increased healthcare costs. The nutritional assessment in pediatric CKD employs a comprehensive, multidisciplinary approach, considering disease-specific factors, growth metrics, and dietary habits. The prevalence of malnutrition, as identified through diverse tools and guidelines, underscores the necessity for regular and vigilant monitoring. Nutritional management strategies seek equilibrium in calorie intake, protein requirements, and electrolyte considerations. Maintaining a well-balanced nutritional intake is crucial for preventing systemic complications and preserving the remaining kidney function. The nuanced landscape of enteral nutrition, inclusive of gastrostomy placement, warrants consideration in scenarios requiring prolonged support, with an emphasis on minimizing risks for optimized outcomes. In conclusion, the ongoing challenge of managing nutrition in pediatric CKD necessitates continuous assessment and adaptation. This review underscores the significance of tailored dietary approaches, not only to foster growth and prevent complications but also to enhance the overall quality of life for children grappling with CKD.

**Graphical abstract:**

**Figure:**
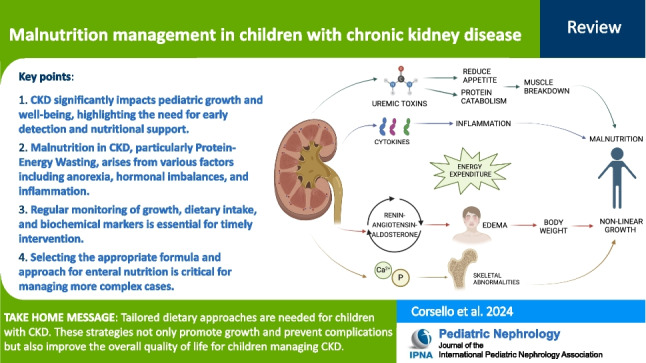
A higher resolution version of the Graphical abstract is available as Supplementary information

**Supplementary Information:**

The online version contains supplementary material available at 10.1007/s00467-024-06436-z.

## Introduction

The complex interplay between childhood chronic disease and nutritional well-being represents a multidisciplinary problem that requires comprehensive understanding [1]. Recent studies have uncovered an alarming trend of nutritional imbalances, which are particularly pronounced among children with chronic illnesses [2]. Among these patients, chronic kidney disease (CKD) occupies an important place and has a profound impact on a relatively large part of the pediatric population [3]. CKD can be diagnosed when a combination of markers of kidney damage, including albuminuria and abnormal urinary sediment, persists for more than three months [4].

Childhood kidney disease encompasses a variety of conditions, each presenting unique challenges and implications for both the affected individuals and the healthcare system. Epidemiological data provide information about the prevalence and patterns of these diseases and help physicians identify kidney diseases with an increased risk of progression to CKD.

Since up to 50% of patients with kidney failure may experience significant growth impairment, early detection and adequate nutritional support are critical to ensure appropriate development and long-term well-being [5]. This review attempts to summarize the various factors contributing to malnutrition in these patients and to suggest tailored interventions designed to mitigate the risk of adverse clinical outcomes.

## CKD and malnutrition in the pediatric age group

A number of diseases can cause kidney failure, also referred to as CKD stage 5 [6, 7]. According to the 2023 Report – US Renal Data System (USRDS) database [8], the congenital anomalies of the kidney and urinary tract (CAKUT) are the leading cause (28.3%) of kidney failure, even if prevalence rates vary depending on geographic and demographic factors [7]. The second leading cause is represented by glomerulonephritis (21.2%) [9]. Although hereditary nephropathies are rare in individual cases, overall, they account for a significant proportion of pediatric kidney failure cases and require careful attention in early detection and treatment. Other causes of kidney failure in the pediatric age group include tubulointerstitial diseases, acquired obstructive uropathy, transplant complications, hypertensive or large vessel disease, and tumors [8].

Dialysis becomes a crucial consideration in cases where chronic kidney failure reaches an advanced stage, generally with GFR < 15 mL/min/1.73 m^2^ [10]. However, the indications for initiating dialysis in children involve several factors, including fluid and electrolyte imbalances, uremic symptoms, growth retardation, and metabolic disorders [11].

In the field of CKD and dialysis, the intricacies of malnutrition go beyond a general understanding and address the specific nuances of various pathological manifestations. Patients face an unpredictable number of factors that may contribute to the risk of malnutrition, increasing the complexity of their conditions. The consequences of chronic malnutrition in this context are far-reaching, ranging from developmental and social consequences to multi-organ dysfunction, long hospital stays, and increased healthcare costs [2]. In patients with CKD, multiple factors contribute to the development of malnutrition. The pathogenic mechanisms underlying malnutrition in CKD are complex and involve a combination of physiological changes, including gastrointestinal symptoms related to nephropathy, loss of appetite, dietary quality, albumin depletion, reduced nutrient intake, hormonal imbalances, metabolic disturbances, chronic inflammation, increased catabolism, and dialysis-related issues [12–15]. For the sake of clarity, this review will focus on three key factors in CKD-related malnutrition: protein-energy wasting, hormonal imbalances, and inflammation.

### Protein-energy wasting

Patients with CKD are at substantial risk for malnutrition, characterized by protein-energy wasting (PEW) and micronutrient deficiency. Many of the factors may cause or worsen other factors. For instance, the chronic inflammatory state induces anorexia, decreases protein and caloric intake, and reduces the synthesis of albumin leading to PEW and hypoalbuminemia [16].

Although there is no accepted definition of pediatric PEW, it is manifested by low serum albumin levels, reduced body mass index (BMI), reduced muscle mass, and impaired growth [16]. This condition is exacerbated by several factors typical of CKD, including chronic inflammation, acidosis, impaired insulin signaling, and abnormal appetite regulation [17]. The complicated relationship between CKD and PEW is further illustrated by the results of the Chronic Kidney Disease in Children Study (CKiD) study, which showed a widespread prevalence of PEW diagnosis in children with CKD, ranging from 7 to 20% [18].

Diagnostic criteria for PEW in pediatric patients, adapted from adult guidelines, were established by CKiD [19]. These criteria include inadequate linear growth, BMI, or Mid-Upper Arm Circumference (MUAC) less than 5% for height age, and a BMI or MUAC change of 10% or more between the first and second visits in non-obese children. It’s crucial to acknowledge that PEW in children with CKD is associated with an elevated risk of hospitalization or emergency room visits and a diminished quality of life, and the inflammation linked with PEW is considered a cardiovascular risk factor. An abnormal hormonal environment, indicated by imbalances in leptin and ghrelin, is believed to contribute to PEW development in CKD patients, although the precise role of these factors is not completely understood [19].

Thus, PEW may not be due solely to inadequate nutrient intake but rather may be caused by a complex interplay of factors that contribute to maladaptive responses in the body. The resulting malnutrition can then lead to stunted growth and reduced muscle mass. In addition, malnutrition may increase the already increased risk of comorbidities of CKD, including cardiovascular disease, bone abnormalities, and immune deficiency, thereby affecting overall quality of life [20].

### Hormonal imbalances

In CKD, there is a complex interplay of physiological disorders that directly and indirectly contribute to the development of malnutrition. Furthermore, emerging evidence suggests that inflammation plays a critical role in altering insulin signaling and triggering muscle wasting and impaired glucocorticoid insulin-like growth factor 1 (IGF1) production [21]. Anorexia is common in children with CKD and is itself a key cause of malnutrition. Although basal metabolic rates reported in the literature generally appear to be comparable to those in healthy peers, anorexia and growth appear to worsen as the glomerular filtration rate (GFR) decreases [22]. The origins of this phenomenon may be related to impaired modulation of appetite hormones such as ghrelin and leptin [23, 24].

CKD disrupts the hormonal balance responsible for regulating nutrient homeostasis. Dysregulation of the renin–angiotensin–aldosterone system affects sodium and water balance and can lead to fluid retention and edema, which can impair accurate assessment of nutritional status by affecting body weight [25, 26]. The imbalance of calcium, phosphorus, and vitamin D affects bone health and growth and leads to skeletal abnormalities [27].

### Inflammation

Indirectly, the effects of kidney damage impact various body systems and contribute to a multifaceted nutritional challenge. Furthermore, eventual chronic inflammation, which can often occur in these patients as an inherent feature of CKD, promotes the release of pro-inflammatory cytokines such as IL-6 and TNF-alpha [28, 29]. These cytokines maintain the catabolic state, suppressing appetite and further increasing the risk of malnutrition. The increased oxidative stress associated with CKD increases cellular damage and weakens antioxidant defenses, potentially impairing nutrient utilization.

Both kidney-specific factors and systemic consequences play a role, which together shape the nutritional landscape of affected children [30]. Kidney damage in CKD directly triggers a cascade of mechanisms that impair the body’s ability to maintain proper nutritional status. Impaired kidney function impedes the excretion of waste products, leading to the accumulation of uremic toxins [31]. These toxins, including urea and creatinine, disrupt appetite regulation, alter the sense of taste, and cause nausea and vomiting, which impair food intake [32]. The altered metabolism of amino acids and proteins can exacerbate this challenge and lead to net protein catabolism [33]. The result is muscle breakdown and loss of lean body mass, leading to weight loss and reduced functionality.

In addition, due to the described increased oxidative stress, CKD can disrupt the composition of the intestinal microbiota and lead to dysbiosis [34, 35]. This imbalance could even affect gut integrity, nutrient absorption, and metabolism of bile acids and short-chain fatty acids. Gut dysbiosis also contributes to systemic inflammation and may also indirectly impact appetite-stimulating hormones such as ghrelin [36].

**Fig. 1:**
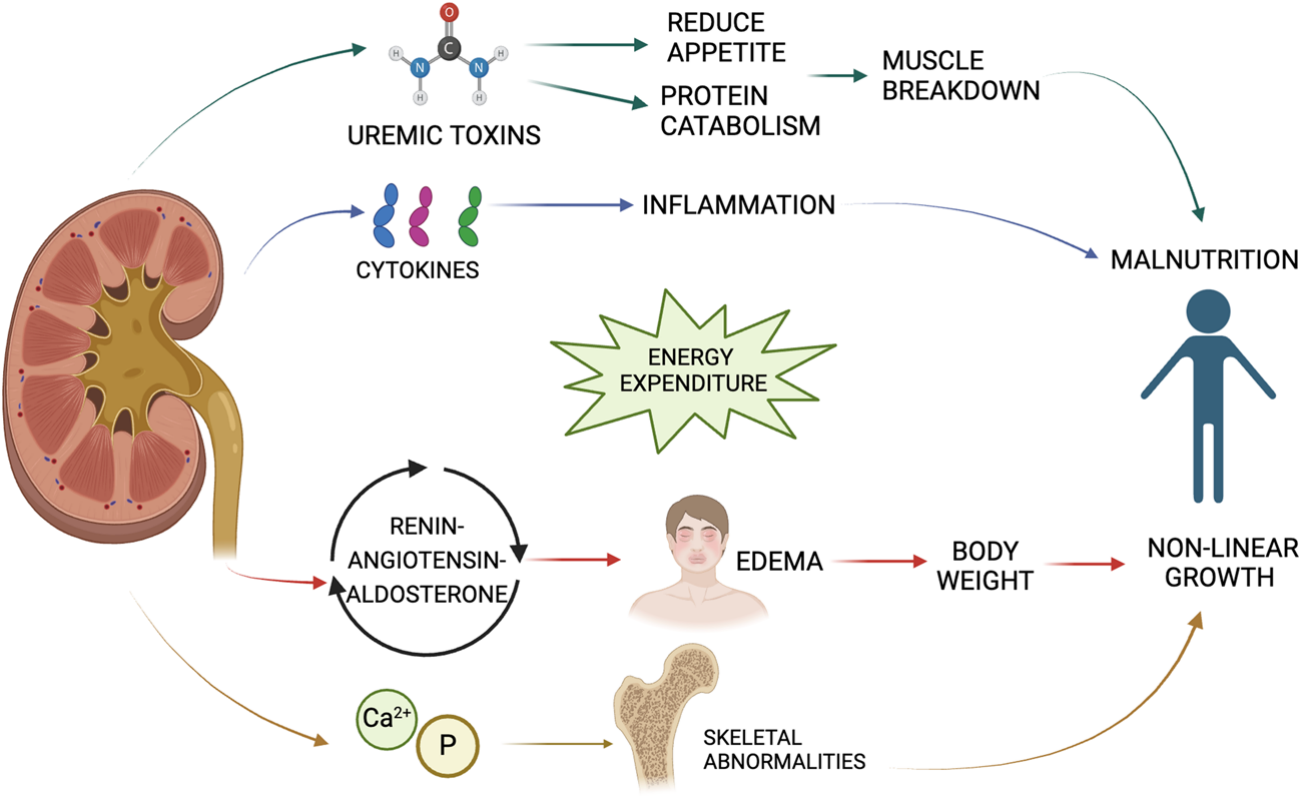
Pathophysiology of malnutrition in CKD

Figure 1 represents an overview of the pathophysiology of malnutrition in patients with CKD.

## Nutritional assessment in pediatric CKD

Nutritional assessment of children with CKD must include a multidisciplinary assessment that considers various pathogenetic aspects of the disease and goes beyond dietary calorie/protein intake to include the broader context of CKD-induced physiological and metabolic disorders.

Dietary management should be tailored to each patient based on their CKD stage and kidney replacement therapy modality. The goal is to ensure adequate calorie and protein intake while restricting protein, potassium, and phosphorus as necessary. Therefore, a comprehensive assessment of micronutrient intake is essential at the outset of patient management.

In addition, other risk factors such as young age, early onset of kidney failure, longer duration of dialysis, medication use (e.g., prolonged treatment with corticosteroids), fluid requirements, proteinuria, and comorbidities such as congenital heart defects or neurological disabilities may contribute to an increased incidence of malnutrition and PEW.

These factors underscore the need for comprehensive data collection to elucidate their complex relationships with the nutritional status of children with CKD. Currently, available guidelines offer guidance on how to assess daily requirements in children, but the distinction between malnourished and well-nourished children can be difficult because of the fluid overload and heterogeneous distribution of muscle mass and adipose tissue [37, 38].

This aspect requires very close collaboration between the nutritionist/dietitian and the pediatric nephrologist to find a meeting point for the correct treatment of the patients. Current assessment toolkits include anthropometric data and dietary intake assessments, as well as comprehensive insights into growth trajectories and dietary habits [21, 38], the same as those described for other diseases such as cancer [39].

As suggested by the Pediatric Renal Nutrition Taskforce, anthropometric measurements such as weight, height (or length), and head circumference should be the primary assessment tools for children with CKD [40]. It is important to note that the presence of edema may indicate that the measured weight overestimates the child’s euvolemic weight. Determining the child’s euvolemic weight is crucial, particularly for those with oliguria anuria, or active nephrotic syndrome, as fluid retention can lead to inaccuracies in weight assessment. These measurements should be regularly plotted on growth charts to calculate BMI and z-scores. Additionally, DEXA and Bioelectric Impedance Analysis (BIA) may be used for nutritional assessment, although these methods are more expensive and less suitable for routine use. MUAC measurement, moreover, can be a quick and easy assessment tool, less affected by fluid status/overload than body weight, even if its sensitivity and specificity still need to be evaluated in children with CKD [40, 41].

During nutritional assessment, evaluating dietary quality is essential for identifying macro and micronutrient deficiencies. Biochemical analyses, including serum albumin, albumin, total proteins, transferrin, creatinine, hemoglobin, lymphocyte count, cholesterol, triglycerides, and retinol-binding protein, provide valuable insights into nutritional status [42]. However, none of these markers are sensitive or specific enough to exclusively diagnose PEW [37].

Based on these premises, the traditional focus of nutritional assessment in children with CKD is primarily on the detection and avoidance of malnutrition. The CKiD study found unintentional weight loss that worsened as CKD progressed [18, 43, 44]. Those who experienced significant weight loss were also more susceptible to developing kidney failure, with most severe cases exhibiting multiple signs of fragility, impaired muscle mass, fatigue, and inflammation, with a resulting increased risk of infection or hospitalization. Additionally, data from the same cohort showed that 33% of participants were classified as either overweight or obese, which persisted even after kidney transplantation.

The National Kidney Foundation’s Kidney Disease Outcomes Quality Initiative (KDOQI) guidelines emphasize the need for more frequent assessment of nutritional and growth parameters compared to healthy peers [45, 46]. For children < 1 year old with CKD stage 2–4, screening every 2 to 6 weeks is recommended. The rhythm shifts in children older than 1 year, with assessment every 1–3 months, and progresses in children older than 3 years, with monitoring recommended every 1–6 months depending on the severity of CKD.

The Pediatric Renal Nutrition Taskforce has recently recommended utilizing a nutrition-focused physical examination as a validated tool for anthropometric assessment in the general pediatric population, using the World Health Organization (WHO) growth charts for all ages or country-specific charts, if available [40]. They also suggest the use of waist-high ratio and grip strength to determine an increased risk of poor outcomes and malnutrition. Incorporating BMI categorization is proving to be a valuable tool for identifying overweight and obese children and promoting a proactive approach to nutritional management. Assessment of food intake and diet history are crucial aspects that can be achieved through methods such as food frequency questionnaires or food reminders, facilitated by dedicated electronic applications [47]. Currently, the 3-day diet history stands as the gold standard for clinically assessing nutritional intake in children with CKD [40]. However, it is crucial to explore the potential of innovative methods, such as food apps, e-mailed records, and pictorial food records, to enhance the accuracy and efficiency of dietary intake assessment compared to the traditional 3-day diet history.

The cornerstone of nutritional management is therefore to prevent both stunting and wasting while avoiding obesity caused by overnutrition. This complex balance requires a personalized and educational approach tailored to each patient’s individual needs and circumstances. Furthermore, the surveillance process needs to be tailored to specific CKD diseases, as glomerular CKD cases require increased vigilance against PEW as they tend to result in marked weight loss [12].

Strategies for identifying and diagnosing malnutrition (Table 1) should include a growth assessment, based on specific charts for age, together with the assessment of food intake, including quantity and type of food, and serum biochemical analyses. Finally, the degree of malnutrition should be classified as mild, moderate, or severe based on the interpretation of z-scores, the decline in z-scores over time, and the percentage change in weight compared to the norm [48].

**Table 1:** Strategies for identifying and diagnosing malnutrition

Growth assessment	
0–2 years	WHO growth charts z-score for weight (weight gain should be compared with WHO standards for average grams of weight gain per day) z-score for length z-score for weight-for-length z-score for head circumference
> 2 years	WHO growth chart for all ages or country-specific growth charts, if available Plot BMI or weight-for-length on centile growth charts
Assessment of food intake	Food frequency questionnaires (e.g., 3-day diet history) Food reminders
Biochemical analyses	Albumin, Prealbumin, and Total proteins Electrolytes Transferrin and Ferritin Creatinine Hemoglobin and lymphocyte count Cholesterol and triglycerides Retinol-binding protein

## Nutritional management

In the complex interplay between evidence-based guidelines and personalized intervention, the landscape of nutritional management for pediatric CKD patients represents a dynamic area that not only promotes better nutritional outcomes and overall growth but also improves the quality of life and well-being of children and adolescents. Calorie and protein intake is a cornerstone of achieving a balanced diet [5]. The assessment of the caloric needs of CKD patients takes into account the same factors as their healthy counterparts, such as body size, physical activity, and basal metabolism [37]. Especially in the first two years of life, a special focus on adequate nutrition is crucial for optimal growth and development. In this context, breast milk or age-appropriate formula, possibly enriched with calorie supplements such as maltodextrins, can serve as an essential source of nutrition for infants [49]. Moreover, careful attention should be paid to fluid balance, especially during dialysis, to avoid high blood pressure and electrolyte imbalances. Achieving a balance between fluid restriction and adequate hydration is critical, as intravascular fluid imbalances can precipitate cardiovascular complications, increasing morbidity and mortality risk.

### Energy

The energy requirements of CKD patients are generally like those of healthy children of the same age. However, adjustments to energy intake should be made based on weight fluctuations. For children on peritoneal dialysis, energy expenditure related to dialysis must be considered, necessitating increased energy requirements in some patients. Changes in caloric intake should also consider age-related modifications and lifestyle changes, such as alterations in physical activity [45]. However, acute illnesses can influence estimated energy requirements, requiring careful monitoring and dietary adjustments. In cases where increased energy intake is necessary without a corresponding increase in protein consumption, fortification methods involving carbohydrate supplements and/or fat emulsions may be utilized.

### Proteins

Effective management of CKD nutritional needs necessitates careful monitoring and adjustment of protein intake, as it plays a crucial role in determining mortality rates. A low-protein diet is rarely recommended [50]. To promote optimal growth protein intake should be at the upper end of recommendations (1–1.2 g/kg/day). Avoiding dietary protein restriction in early-stage CKD children is advisable to mitigate the risk of malnutrition, poor growth, and PEW. If protein intake needs to be reduced it should not fall below the minimum amount (0.8 g/kg/day) [38]. For non-critically ill children with CKD, the initial suggested protein intake should be based on “dry” or euvolemic weight [51]. Critically ill children, however, may require increased protein intake above the standard to limit negative protein balance. In cases of very elevated blood urea nitrogen levels, adequate energy intake should be ensured first, followed by temporary lowering of protein intake towards the lower end of the recommended intakes [38]. However, compromising protein intake persistently to lower urea nitrogen levels or postpone kidney replacement therapy initiation is strongly discouraged.

Children requiring dialysis for advanced CKD have increased protein requirements that compensate for losses during hemodialysis and peritoneal dialysis, which almost double in infants. Protein intake is crucial and should be at least 100% of the recommended daily intake (DRI), while severe patients on dialysis could warrant up to 140% of the DRI for stages 2–3 [38, 45, 46]. It is crucial to find the right balance as excessive protein intake, especially animal proteins, can lead to uremic toxicity [52, 53]. Sources such as lean meat, poultry, fish, eggs, dairy products, and plant-based proteins (e.g., beans, lentils, tofu) should generally be preferred [38, 54]. The consumption of animal protein is associated with an increased risk of hyperfiltration, albuminuria, and kidney failure [55]. On the other hand, the consumption of plant proteins provides protective effects for both the kidneys and cardiovascular system [56].

### Carbohydrates and lipids

Patients with CKD are at increased risk of impaired glucose metabolism and insulin resistance [57, 58]. This makes sugar and carbohydrate intake a significant problem. A diet high in whole grains, fiber, and complex carbohydrates is recommended, while sugary foods and drinks should be limited [37, 38, 59]. Careful monitoring and coordination with endocrinologists are essential for glycemic control.

Due to the increased risk of dyslipidemia and cardiovascular complications, a balanced intake of healthy fats should always be considered, with an emphasis on unsaturated fats while limiting saturated fats and trans fats [38, 60]. Omega-3 fatty acids found in fatty fish have anti-inflammatory properties and may be beneficial [61]. The Pediatric Renal Nutrition Taskforce clinical practice recommendations on obesity and metabolic syndrome do not suggest the routine use of statins and other lipid-lowering agents in children [62].

### Electrolytes and micronutrients

Patients with CKD may waste sodium due to loss or inadequate reabsorption and therefore require sodium supplements while avoiding sodium high concentrations since the risk of developing hypertension is increased in these patients [63]. According to the American Heart Association, approximately 15% of dietary sodium is naturally occurring in foods, 11% is added during cooking, and over 70% comes from processed foods [64]. While the body requires around 500 mg/day of sodium for essential functions such as muscle movement and mineral balance maintenance, excessive intake can elevate the risk of high blood pressure, heart disease, and stroke. The KDOQI guidelines recommend limiting sodium intake to 1500–2300 mg/day for children with CKD who have hypertension or prehypertension [45]. However, it is important to highlight that adhering to a low-sodium diet can be challenging for CKD patients, especially those in the pediatric age group.

Potassium plays a crucial role in maintaining cellular osmolarity, with approximately 2% present in extracellular fluid (3.5–5.0 mEq/L) and 98% in cells (140 mEq/L) [65]. Reduced kidney function can lead to elevated serum potassium levels, typically manifesting when GFR falls to 15–20 mL/min/1.73 m^2^. While potassium-containing foods are not automatically restricted in the diet, limitations are imposed if hyperkalemia is present. Breast milk or infant formula serves as the primary potassium source for infants, while children and adolescents obtain potassium from various natural dietary sources such as milk, potatoes, vegetables, cereals, fruits, and meat [66].

Calcium and phosphorus are the primary electrolytes responsible for maintaining optimal bone and mineral balance, thus preventing skeletal issues and supporting proper growth in children, especially in CKD, where disruptions in mineral metabolism occur. Natural dietary sources of calcium and phosphorus include dairy products (e.g., cow’s milk, cheese, yogurt), breast milk, infant formulas, grains, meat, and meat derivatives. Guidelines recommend regular dietary assessment of calcium and phosphorus content in children with kidney disease, with adjustments made to maintain serum levels within the appropriate range for their age [67]. Children with CKD should aim to consume 100% to 200% of the age-appropriate DRI for calcium, typically ranging from 210 to 1300 mg/day, while phosphorus intake should be limited to 80% of the DRI to effectively manage hyperphosphatemia [64].

Magnesium is a vital mineral that regulates various bodily functions, serving as an enzymatic cofactor involved in protein and DNA synthesis, energy production, and antioxidant protection. CKD patients are at risk of developing hypomagnesemia due to dietary restrictions on potassium, which may inadvertently reduce magnesium intake [68].

For children with pre-dialysis CKD, guidelines suggest increasing intake of B vitamins or vitamin C either through diet or supplements if intake is lower than recommended for healthy children [69]. Additionally, children undergoing dialysis may benefit from higher vitamin C intake, although the safe amount to ingest to avoid systemic oxalosis is uncertain. Moreover, water-soluble multivitamin supplements, particularly vitamins C, B6, and folate, may be necessary for children on dialysis due to dialysate losses [69]. Folate and vitamin B12 supplementation may also be required for children with macrocytic anemia and biochemical evidence of deficiency. However, routine supplementation of vitamins A, E, and K is discouraged unless there is a comorbidity predisposing to deficiency.

Anemia is a common complication in CKD, primarily due to iron deficiency, resulting from factors such as reduced erythropoietin production, shortened red blood cell lifespan, inflammation, hemolysis, blood loss, and nutritional deficiencies [70]. Low zinc levels may increase the risk of progressing to kidney failure and protect against phosphate-triggered calcification in CKD patients. Additionally, children with CKD are at risk of copper deficiency, which can result from dietary restrictions, malnutrition, and gastrointestinal malabsorption.

## Enteral nutrition and gastrostomy options

Enteral nutrition (EN) may be considered if the child’s nutritional needs cannot be fully met. If there are no specific contraindications, a standard polymer formula can be used initially (for children from 1 year old). If a reduction in the volume of enteric feeds is necessary, a high-calorie formula (1.5 kcal/ml) may be used [71]. According to the Pediatric Renal Nutrition Taskforce, to accommodate fluid restrictions or mitigate issues like vomiting and gastroesophageal reflux, the nutritional content of infant formulas can be concentrated into a smaller volume [38]. Typically, standard infant formulas are reconstituted to around a 13% concentration, providing 67 kcal and 1.3 g of proteins per 100 ml. A gradual increase in this concentration by 1–3% daily can be performed, potentially up to 20% (20 g of powder per 100 ml of water), enhancing the overall energy density to up to 1 kcal per ml. However, it is important to implement this concentration process gradually to ensure the infant’s tolerance, as an abrupt increase in osmolality can lead to side effects such as diarrhea, vomiting, and gastroesophageal reflux [72]. In addition, the concentration of the formula increases the solute load on the kidneys and may lead to excessive absorption of minerals and vitamins such as phosphate and potassium, which requires careful monitoring.

Other options may include real food-based formulas, which are becoming increasingly popular among parents and caregivers of long-term tube feeding patients. Real food-based formulas are enteral formulas (1.2 kcal/mL) with real food ingredients such as a milk-based mixture of peas, green beans, peaches, carrots, and chicken. They may also help relieve gastrointestinal symptoms such as constipation, reflux, and feelings of choking [73]. However, to date, there is no data on the use of these formulas in children with CKD.

Even if dietary supplementation with tube feeding may lead to nutritional imbalance if managed incorrectly, it has been shown that gastrostomy feeding improves both weight and BMI without leading to obesity [74]. It is then necessary to carry out continuous clinical and instrumental follow-up to check the clinical status, growth, fluid balance, electrolyte, macronutrient, and vitamin levels in these patients. The choice of modality carries risks and complications, particularly in peritoneal dialysis patients who are at increased risk of infection. In most cases of mild or absent dysphagia, the goal of tube feeding is to restore full oral nutrition after transplantation, which requires constant parental support to maintain constant oral motor function. The routes of administration for EN are consistent with those of other patients, including the nasogastric tube and percutaneous endoscopic gastrostomy (PEG). The nasogastric tube represents the first access route that should be used for EN [75]. The nasogastric tube insertion procedure is straightforward, easy to learn, and generally carries minimal risk of peritonitis for pediatric patients undergoing peritoneal dialysis [76]. However, feeding through a nasogastric tube has several disadvantages, including the need to repass the tube frequently and the risk of complications such as food aspiration, reflux esophagitis, impaired oromotor skills, and changes in the child’s appearance. Of particular concern is the possibility of tube obstruction and inhalation of formula, which could be fatal if unsupervised, continuous feeding occurs overnight at home.

When prolonged support (> 4–6 weeks) is required or there is a need to bypass the nasopharynx, gastrostomy (PEG, radiologically inserted or surgical) can be considered. However, gastrostomy procedures also have their own set of complications. These include issues with tube functionality, skin damage due to leakage around the site, and less common but serious complications such as damage to nearby organs, accidental formation of a gastrocolic fistula, leakage of contents into the abdominal cavity, and peritonitis [77].

During peritoneal dialysis, there is a risk of bleeding and/or peritonitis during PEG insertion, as well as the possibility of unsuccessful placement. Because of the serious risks associated with peritonitis, the Pediatric Renal Nutrition Taskforce recommends inserting a gastrostomy device whenever possible before placing a peritoneal dialysis catheter [76]. For a child already receiving dialysis, the taskforce recommends considering an open surgical gastrostomy, or PEG, with laparoscopic assistance to minimize complications [78, 79].

When intragastric feeding is contraindicated, jejunal enteral access could be considered by a nasojejunal tube, a jejunal tube introduced through a gastrostomy, or surgical transcutaneous jejunostomy. A jejunal tube needs to be positioned distal to the Treitz ligament to prevent the retrograde filling of a dysfunctional stomach.

However, it is important to underline that PEG and J-PEG could be contraindicated for patients with PD for increased risk of peritonitis. To reduce this risk, in case of necessity, some evidence suggests the temporary transition to parenteral nutrition [71], without overlooking the risks associated with parenteral nutrition itself and the necessary presence of a central venous catheter.

## Conclusions

The management of malnutrition in children with CKD is a continuous challenge that necessitates ongoing assessment and adaptation of the diet as the child ages and CKD advances. Balancing the requirements for calories, protein, and electrolytes is essential for fostering growth and facilitating weight gain in children with CKD. The goals of a proper and balanced nutritional intake are to control symptoms and prevent complications, especially uremia and disturbances in Ca/P metabolism, to promote adequate growth, and to preserve residual kidney function. The diet should be tailored to each patient, taking into consideration the underlying pathology, the stage of CKD, and the modality of kidney replacement therapy. The vulnerability of infants to growth failure underscores the criticality of ensuring adequate nutrition during the first year of life. In situations where oral intake is scarce, thoughtful consideration should be given to the implementation of enteral tube feeding.

## Supplementary Information

Below is the link to the electronic supplementary material.

Graphical abstract (PPTX 424 kb)
