# Supplementary material for: Malnutrition management in children with chronic kidney disease
Source: Pediatr Nephrol. 2024 Jul 2;40(1):15–24. doi: 10.1007/s00467-024-06436-z (PMC11584524; doi:10.1007/s00467-024-06436-z)
Supplement: Supplementary file 1 — Graphical abstract (PPTX 424 kb) [file 467_2024_6436_MOESM1_ESM.pptx]

## Slide 1
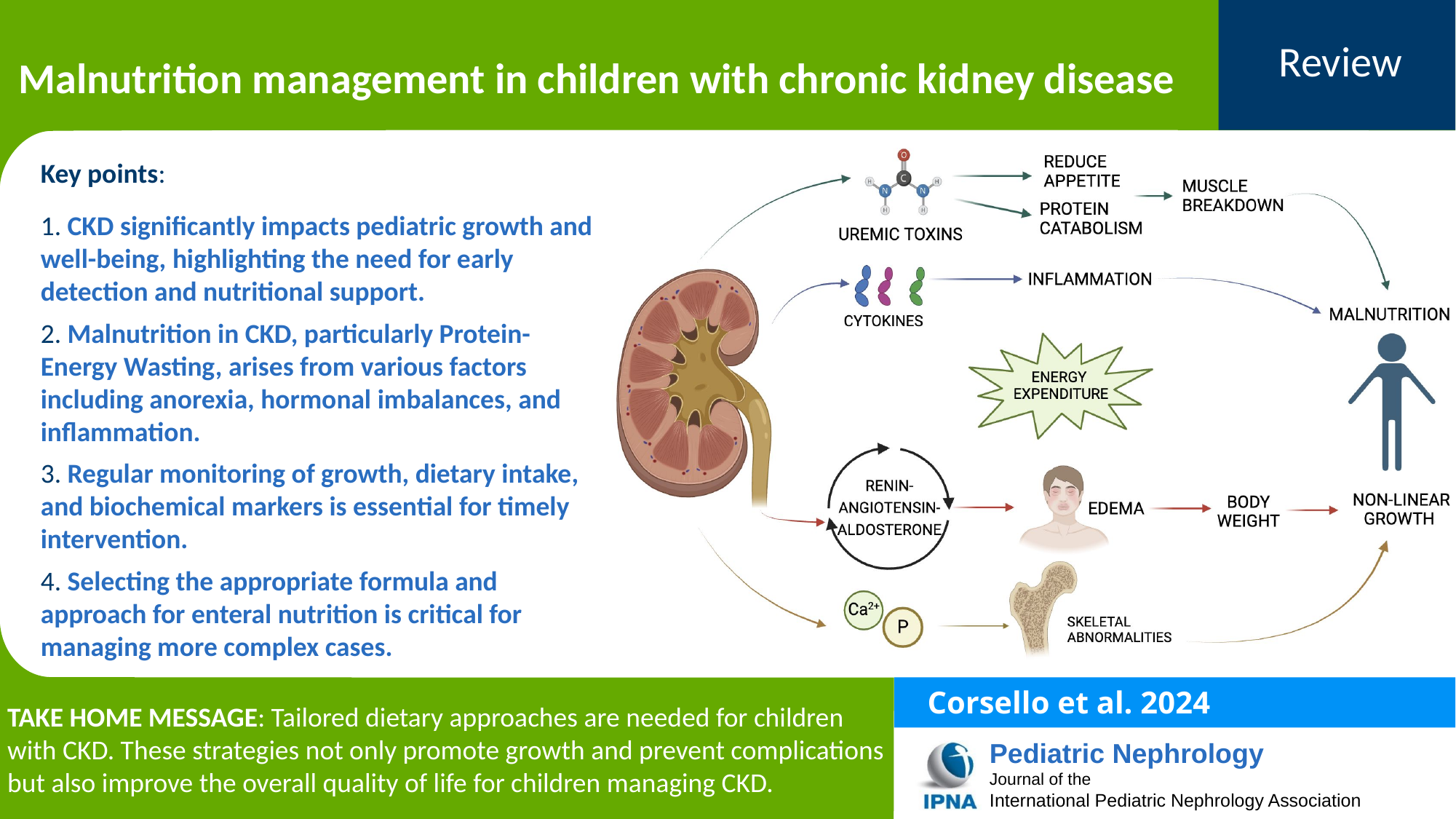

Malnutrition management in children with chronic kidney disease
Key points:
1. CKD significantly impacts pediatric growth and well-being, highlighting the need for early detection and nutritional support.
2. Malnutrition in CKD, particularly Protein-Energy Wasting, arises from various factors including anorexia, hormonal imbalances, and inflammation.
3. Regular monitoring of growth, dietary intake, and biochemical markers is essential for timely intervention.
4. Selecting the appropriate formula and approach for enteral nutrition is critical for managing more complex cases.
Corsello et al. 2024
TAKE HOME MESSAGE: Tailored dietary approaches are needed for children with CKD. These strategies not only promote growth and prevent complications but also improve the overall quality of life for children managing CKD.
